# Supplementary material for: Differential and Cultivar-Dependent Antioxidant Response of Whole and Fresh-Cut Carrots of Different Root Colors to Postharvest UV-C Radiation
Source: Plants (Basel). 2023 Mar 13;12(6):1297. doi: 10.3390/plants12061297 (PMC10053824; doi:10.3390/plants12061297)
Supplement: Supplementary file 1 [file plants-12-01297-s001.zip › Supplementary Tables [S1, S2].pdf]

Supplementary Table S1. Root concentration of mayor anthocyanin glycosides in the purple-rooted cultivars Purplesnax and INTA44

		Retention	Purplesnax		INTA44	
Anthocyanin pigment	Abbreviation	time (min)	mg kg^{-1} fw	% of total	mg kg^{-1} fw	% of total
Cy-3-(2”-xylose-6-glucose-galactoside)	Cy3XGG	13.7	8.2 ± 2.0[§]	1.8	0.3 ± 0.2	1.0
Cy-3-(2”-xylose-galactoside)	Cy3XG	14.3	4.3 ± 2.2	0.9	0.6 ± 0.3	2.0
Cy-3-(2”-xylose-6”-sinapoyl-glucose-galactoside)	Cy3XSGG	14.5	20.4 ± 13.6	4.4	16.4 ± 5.1	54.5
Cy-3-(2”-xylose-6”-feruloyl-glucose-galactoside)	Cy3XFGG	15.0	409.2 ± 73.6	88	12.4 ± 2.1	41.2
Cy-3-(2”-xylose-6”-(4-coumuroyl)glucose-galactoside)	Cy3XCGG	15.4	19.9 ± 3.7	4.3	0.4 ± 0.2	1.3
Total			465.0 ± 85.8	100	30.1 ± 6.6	100

[§] Concentration of cyanidin glycosides expressed on the basis of cyanidin equivalents ± std. err.

Supplementary Table S2. Edaphic characteristics of the carrot growing site

Edaphic parameter	Value (interpretation)
pH	7.5
Salinity (dS/m)	1.8 (not saline)
Total Nitrogen (N) (mg/kg)	1050 (high)
Organic matter (%)	5.6
Available phosphorus (P) (mg/kg)	2.7
Exchangeable potassium (K) (mg/kg)	205
C/N ratio	8.6
Texture	Silty loam
Sedimentation volume (cm^3/100g)	112

Soil analyses were performed at the Laboratory of Edaphology, Faculty of Agricultural Sciences, National University of Cuyo, Mendoza, Argentina.
