# Supplementary figures and images for: Differential and Cultivar-Dependent Antioxidant Response of Whole and Fresh-Cut Carrots of Different Root Colors to Postharvest UV-C Radiation
Source: Plants (Basel). 2023 Mar 13;12(6):1297. doi: 10.3390/plants12061297 (PMC10053824; doi:10.3390/plants12061297)

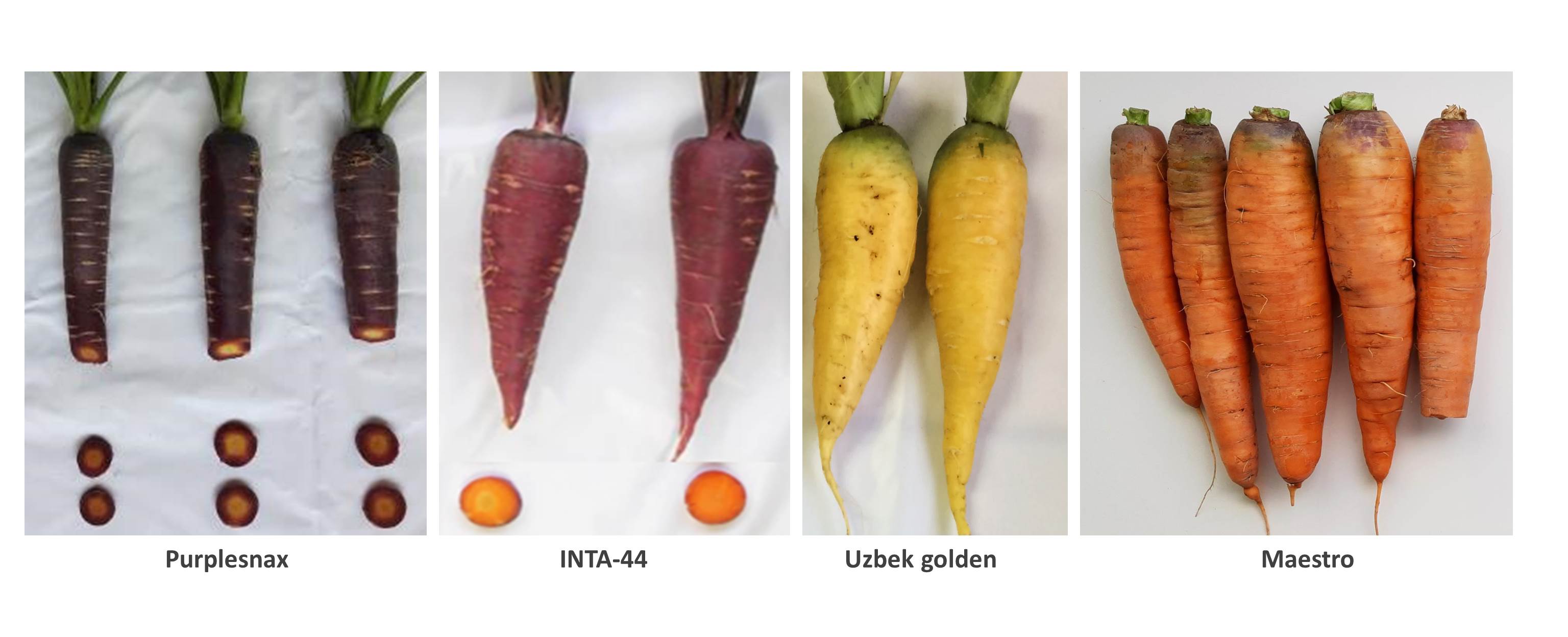

Supplement: Supplementary file 1 [file plants-12-01297-s001.zip › Supplementary Figure S1.jpg]

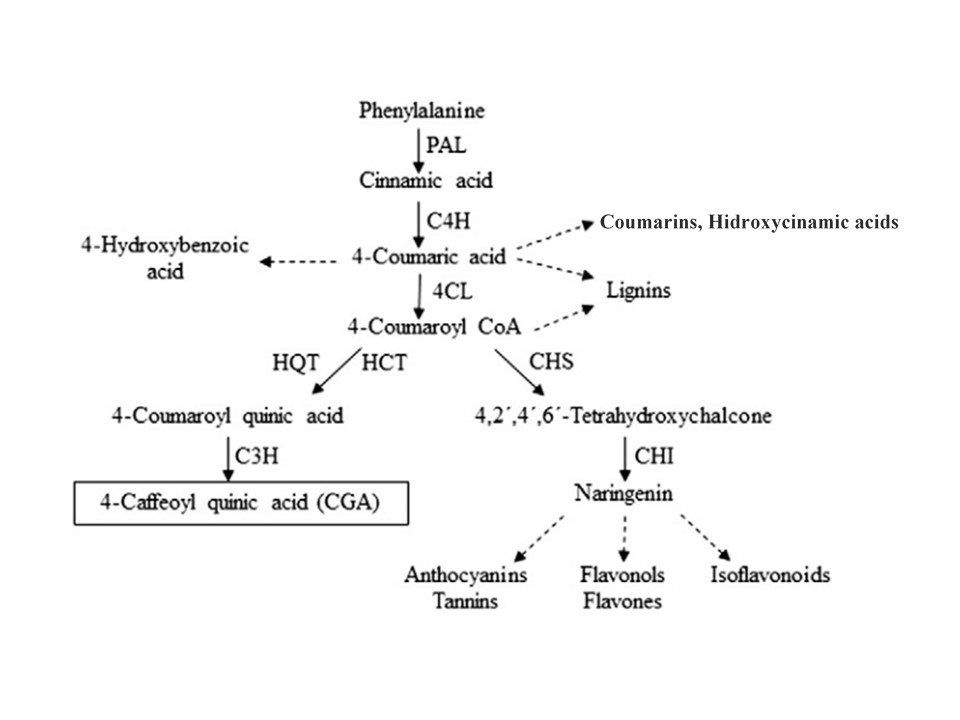

Supplement: Supplementary file 1 [file plants-12-01297-s001.zip › Supplementary Figure S2.jpg]

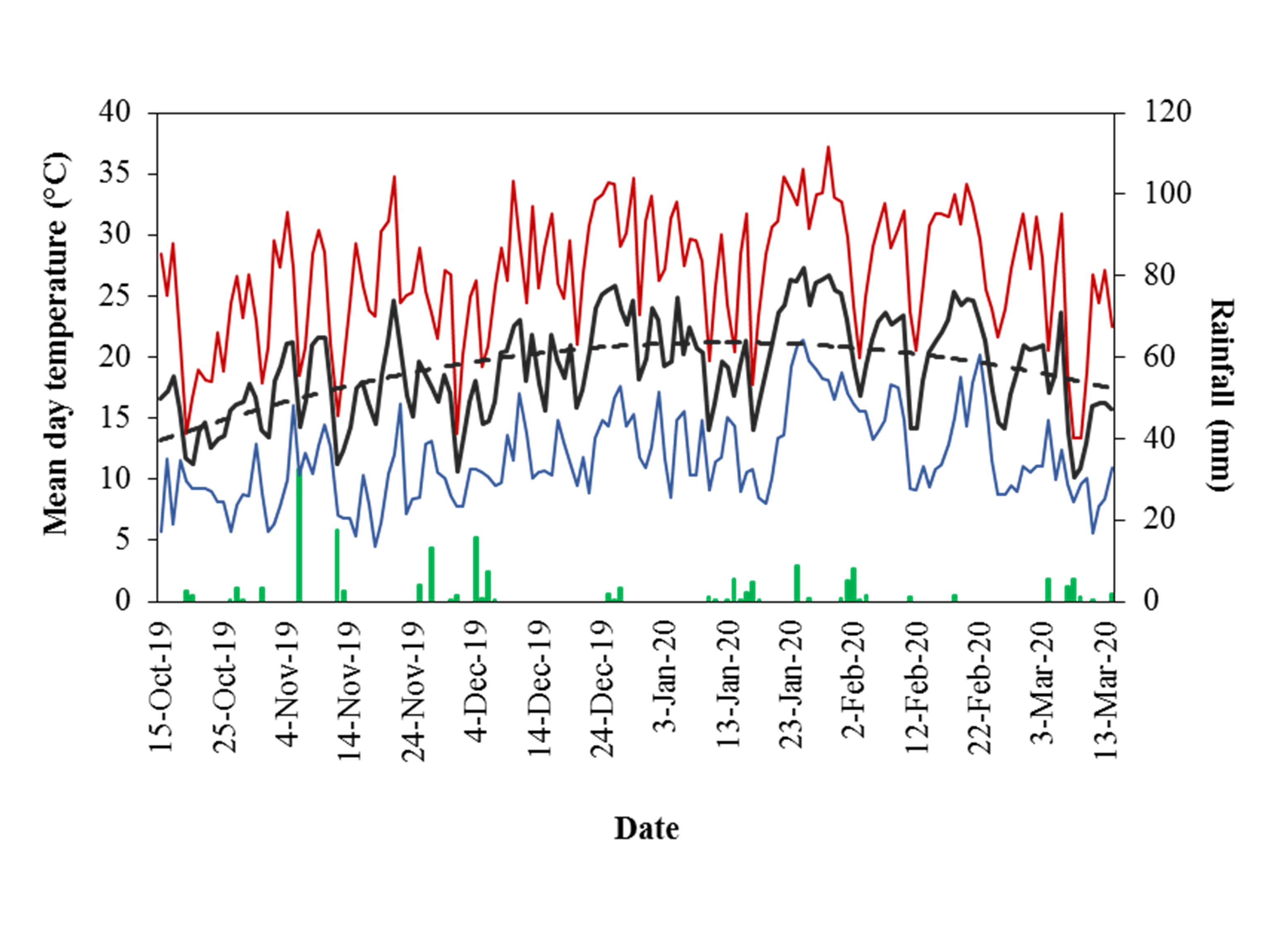

Supplement: Supplementary file 1 [file plants-12-01297-s001.zip › Supplementary Figure S3.jpg]
